# Mortality after transcatheter aortic valve replacement in young multimorbid patients as compared to an age-, gender- and comorbidity-matched background population

**DOI:** 10.3389/fcvm.2025.1600790

**Published:** 2025-06-04

**Authors:** Pernille Steen Bække, Vilhelmas Bajoras, Jawad Butt, Thomas Pilgrim, Nicholas Joseph Montarello, Maurizio Taramasso, Didier Tchetche, Liesbeth Rosseel, Ričardas Kundelis, Kristijonas Česas, Alexander Sedaghat, Jan-Malte Sinning, Rik Adrichem, Mizuki Miura, Magdalena Erlebach, Stephan Windecker, Darren Mylotte, Raj Makkar, Emil Fosbøl, Nicolas Van Mieghem, Ole De Backer

**Affiliations:** ^1^ Department of Cardiology, The Heart Center, Copenhagen University Hospital Rigshospitalet, Copenhagen, Denmark; ^2^ Clinic of Cardiac and Vascular Diseases, Institute of Clinical Medicine, Faculty of Medicine, Vilnius University, Vilnius, Lithuania; ^3^ Division of Cardiology and Vascular Diseases, Vilnius University Hospital Santaros Clinics, Vilnius, Lithuania; ^4^ Department of Cardiology, Bern University Hospital, Inselspital, Bern, Switzerland; ^5^ Clinic of Cardiac Surgery, HerzZentrum Hirslanden Zurich, Zurich, Switzerland; ^6^ Department of Cardiology, Clinique Pasteur, Toulouse, France; ^7^ Hartcentrum Aalst, AZORG, Aalst, Belgium; ^8^ Medizinische Klinik II-Kardiologie, Universitätsklinikum Bonn, Bonn, Germany; ^9^ Department of Cardiology, Cardiovascular Institute, Thoraxcenter, Erasmus University Medical Center, Rotterdam, Netherlands; ^10^ Department of Cardiovascular Surgery, German Heart Centre Munich, Munich, Germany; ^11^ Department of Cardiology, Galway University Hospital, Galway, Ireland; ^12^ Smidt Heart Institute, Department of Interventional Cardiology, Cedars-Sinai Medical Center, Los Angeles, CA, United States

**Keywords:** all-cause mortality, aortic valve stenosis, comorbidity, transcatheter aortic valve replacement, young age

## Abstract

**Introduction:**

Contrary to the current guidelines patients with symptomatic severe aortic stenosis and ≤65 years of age are often referred for transcatheter aortic valve replacement (TAVR). However, the outcome after TAVR in this patient cohort remains unclear.

**Objectives:**

This study aimed to assess the rationale for denial of surgical aortic valve replacement (SAVR) in young multimorbid patients referred for TAVR, to evaluate 3-year all-cause mortality and to compare outcomes with a matched control cohort.

**Patients and methods:**

Retrospective data were collected on all consecutive patients ≤65 years of age with severe aortic stenosis treated with TAVR at 9 centres between 2010 and 2019. The TAVR-population was compared with a 1:4 age-, gender-, and comorbidity-matched population obtained from the Danish National Registries.

**Results:**

The study population consisted of 459 TAVR-recipients and 1,836 matched registry-controls. The main reasons for SAVR denial were prior cardiac surgery (35%), lung disease (30%) and frailty (23%). The 3-year all-cause mortality was 34% in the TAVR-group compared with 8% in the age-, gender- and comorbidity-matched controls with a hazard ratio (HR) of 6.5 (95% CI 4.5–9.6; *P* < 0.001). Patients undergoing TAVR with an active chronic disease (heart failure, lung disease, dialysis) had a 3-year all-cause mortality HR of 1.8–2.4 compared with controls. Overall, 3-year mortality rates in these distinct TAVR-subgroups were high (30%–50%) irrespective of the underlying condition.

**Conclusions:**

Young, multimorbid aortic stenosis patients aged ≤65 years and treated with TAVR between 2010 and 2019 have increased medium-term all-cause mortality compared with an age-, gender- and comorbidity-matched background population.

## Introduction

The European Society of Cardiology (ESC) guidelines on the management of valvular heart disease recommend that transcatheter aortic valve replacement (TAVR) is the preferred treatment for patients with symptomatic severe aortic stenosis (AS) aged ≥75 years (1). On the other hand, the American guidelines consider TAVR as an acceptable alternative to surgical aortic valve replacement (SAVR) for symptomatic severe AS patients aged ≥65 years (2). However, even younger patients are frequently referred for TAVR—despite no randomized controlled data of this treatment option in patients aged ≤65 years. The decision to proceed with TAVR rather than SAVR in this younger, often multi-morbid, cohort of AS patients is made by local Heart Team consensus. This study aimed to determine the basis for TAVR-strategy selection in patients aged ≤65 years, to evaluate the clinical outcomes in terms of 3-year all-cause mortality following TAVR, and to compare the outcomes with an age-, gender- and comorbidity-matched control population without AS.

## Materials and methods

### TAVR study population

The study involved the retrospective collection of data from consecutive patients, aged ≤65 years, undergoing local Heart Team-approved TAVR between 2010 and 2019 within the following TAVR centers: Zurich University Hospital, Zurich, Switzerland; Clinique Pasteur, Toulouse, France; Bern University Hospital, Bern, Switzerland; Cedars-Sinai Medical Center, Los Angeles, USA; Rigshospitalet, Copenhagen, Denmark; Erasmus University Medical Center, Rotterdam, the Netherlands; München University Hospital, München, Germany; Bonn University Hospital, Bonn, Germany; and Galway University Hospital, Galway, Ireland. The study was approved by the data responsible institute [the Capital Region of Denmark (approval number: P-2019-191)] in accordance with the General Data Protection Regulation and by the local Ethics Committees. The investigation conforms to the principles in the Declaration of Helsinki.

### Study concept and primary outcome

**Figure 1:**
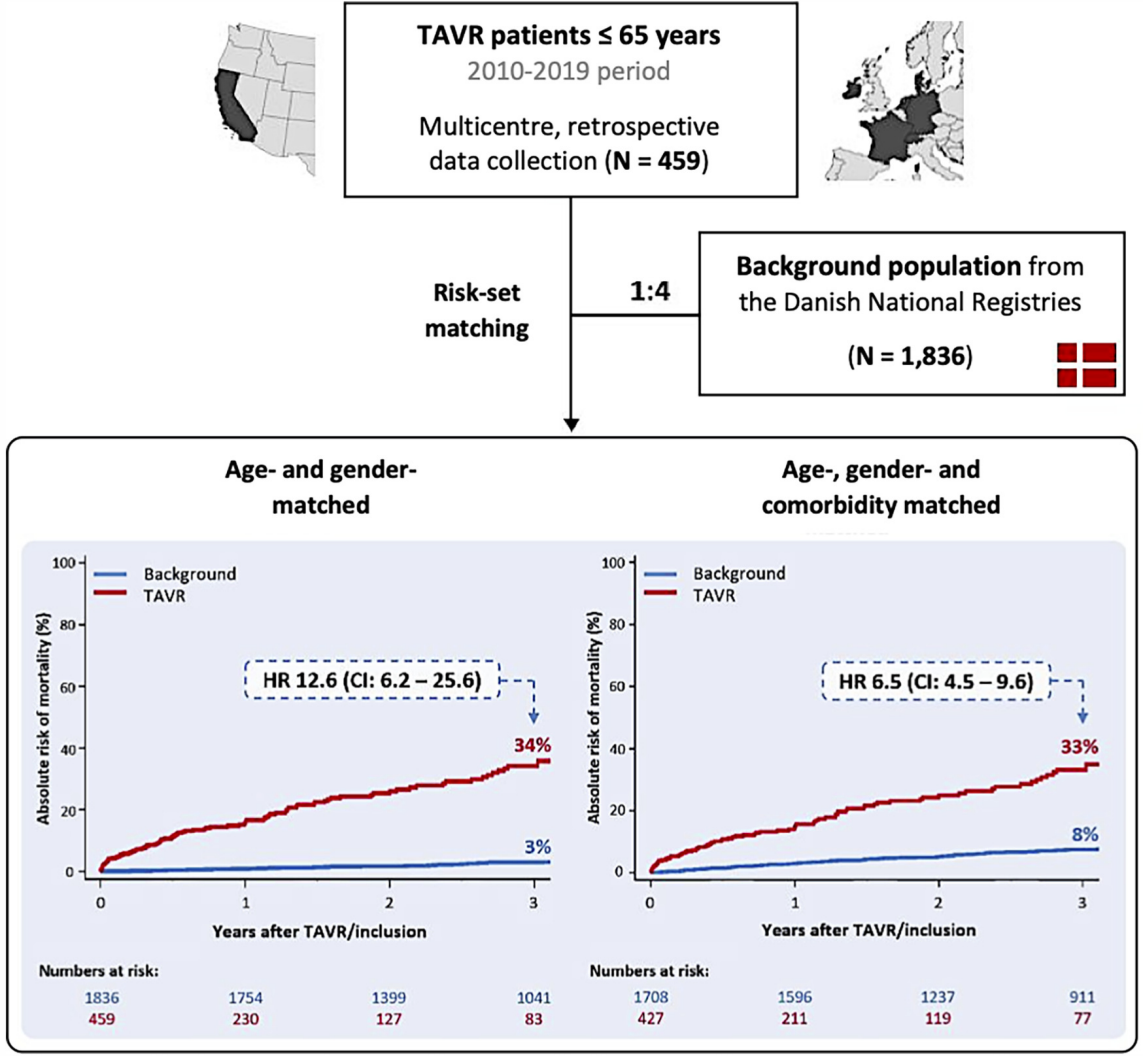
Study concept and primary outcome in the overall study population. HR, hazard ratio; TAVR, transcatheter aortic valve replacement.

This study aimed to conduct a risk-set analysis comparing outcomes between a cohort of patients undergoing TAVR (treated group), and a non-treated cohort of the general population that were similarly matched, derived from the Danish National Administrative Registries (control group). For both cohorts, the index date from which outcomes were measured was set as the date at which TAVR was performed. Using risk-set matching, TAVR patients were matched and compared in a 1:4 ratio with individuals from the Danish National Registries by age (up to a one-year differential), gender, year of the index TAVR procedure (up to a one-year differential), and prior history of ischemic heart disease, chronic heart failure, chronic kidney disease (CKD) and/or renal replacement therapy, chronic obstructive pulmonary disease (COPD), and stroke or transient ischemic attack (Figure 1).

For subgroup analyses, the cohort of patients undergoing TAVR with a specific comorbidity was matched and compared in a 1:4 ratio with age- and gender-matched individuals from the Danish National Registries with the same comorbidity. Two distinct cohorts were evaluated, namely patients with an ‘active chronic disease’, including chronic heart failure, COPD and dialysis, considered active if the individual had been hospitalized due to an exacerbation within 1 year of inclusion into the study, and patients with an ‘inactive prior condition’ including prior cardiac surgery, stroke and Hodgkin lymphoma in remission. The primary outcome of the study was all-cause mortality at 3 years (3).

### Statistical analysis

Baseline characteristics were reported as means with standard deviations for continuous variables and counts with percentages for categorical variables. The absolute risk of all-cause mortality was estimated using the Kaplan–Meier estimator. The rate of all-cause mortality between groups was compared using Cox regression models, stratified by the matching (comparing TAVR cases with their matched controls), and hazard ratios (HR) with 95% confidence intervals (CI) were reported. The level of statistical significance was set at 5%. Data management and statistical analyses were performed with SAS (version 9.4, SAS Institute, Cary, NC, USA).

## Results

### TAVR study population

A total of 459 patients aged ≤65 years undergoing TAVR within the 9 recruiting sites were enrolled in the study. Baseline characteristics of the TAVR population are reported in Table 1. The median age was 61 years (IQR: 57–63 years), 302 patients (66%) were male, and the median calculated Society of Thoracic Surgeons (STS) predicted risk of mortality score was 2.6% (IQR: 1.4%–4.9%). Half of the patients had coronary artery disease (51%) and one-fifth of the patients had atrial fibrillation (21%). The most common non-cardiovascular comorbidities included COPD (35%), diabetes mellitus (32%), and chronic kidney disease (16%, of which 59% requiring dialysis).

**Table 1:** Baseline characteristics and periprocedural/30-day outcomes.

Characteristic/outcome	*N* = 459
Age, years	61 (57–63)
Male	302 (66%)
Arterial hypertension	302 (66%)
Hypercholesterolemia	230 (50%)
Body mass index, kg/m^2^	26 (23–31)
Body mass index >35 kg/m^2^	58 (13%)
Diabetes mellitus	145 (32%)
Coronary artery disease	233 (51%)
Prior myocardial infarction	74 (16%)
Prior percutaneous coronary intervention	133 (29%)
Prior cardiac surgery	159 (35%)
Peripheral arterial disease	125 (27%)
Chronic kidney disease	74 (16%)
Dialysis	44 (10%)
Atrial fibrillation	97 (21%)
Permanent pacemaker	55 (12%)
Prior stroke	48 (10%)
Chronic obstructive pulmonary disease	160 (35%)
Malignancy	72 (16%)
Dyspnea NYHA class 3 or 4	375 (82%)
Left ventricular ejection fraction ≤30%	85 (20%)
Bicuspid aortic valve	66 (14%)
STS-score, %	2.6 (1.4–4.9)
STS-score <4%	308 (67%)
STS-score 4%–8%	93 (20%)
STS-score >8%	58 (13%)
Periprocedural outcomes	
Conversion to SAVR	2 (0.5%)
Second THV implantation	13 (3%)
30 day outcomes after TAVR	
New permanent pacemaker	54 (12%)
Major bleeding	25 (5%)
Major vascular complication	23/400 (6%)
Mortality	19 (4%)
Stroke	7/385 (2%)
Myocardial infarction	2/402 (0,5%)
Acute kidney injury	31/399 (8%)

Values are presented as mean (IQR) or *n* (%). NYHA, New York Heart Association; STS, society of thoracic surgeons; SAVR, surgical aortic valve replacement; THV, transcatheter heart valve.

### Periprocedural and 30-day outcomes after TAVR

The periprocedural and 30-day outcomes and their availability in the population are summarized in Table 1. Permanent pacemaker implantation was required in 54 cases (12%), 13 cases (3%) required second THV implantation and 2 cases were converted to SAVR (0.4%); 25 patients (5%) had major bleeding and 23 (5%)—major vascular complication. A total of 19 (4%) patients died within 30 days of the procedure. A stroke, myocardial infarction and acute kidney injury occurred in 2% 0.5% and 8% of the cases, respectively.

### Reasons for SAVR denial

**Figure 2:**
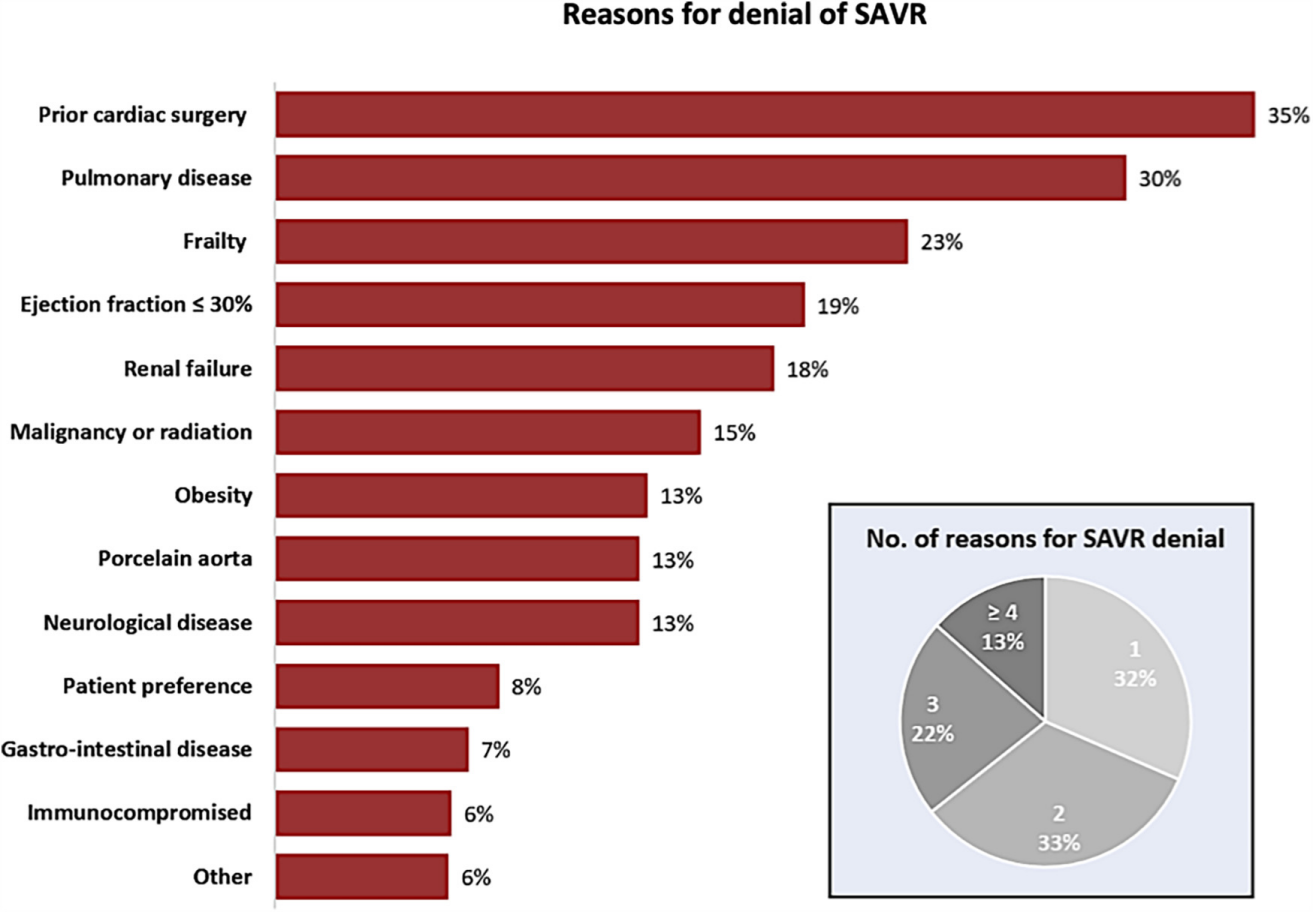
Reasons for denial of SAVR. The rate of patients with a specific comorbidity or condition, mentioned by the local Heart Team, to be a reason for denial of SAVR and referral to TAVR. SAVR, surgical aortic valve replacement; TAVR, transcatheter aortic valve replacement.

The predominant factors documented by the local Heart Teams for not proceeding with SAVR, thereby prompting referral for TAVR, included prior cardiac surgery (35%), the presence of chronic pulmonary disease (30%), frailty (23%), poor baseline left ventricular (LV) systolic function (19%), co-existent chronic kidney disease (18%) and active malignancy or prior chest radiation (15%). Approximately two-thirds of the TAVR cohort had more than a single risk factor for SAVR (Figure 2).

### Matched background population

After risk-set matching, the total study population consisted of 459 patients treated with TAVR and 1,836 patients from the Danish national registry that acted as the control group. At 3 years, all-cause mortality was significantly higher in the TAVR-treated group compared with the control group when matched for age and gender [34.1 vs. 2.9%, *P* < 0.001; 3-year HR 12.6 (95% CI: 6.2–25.6)] and for age, gender and comorbidity [33.1 vs. 7.5%, *P* < 0.001; 3-year HR 6.5 (95% CI: 4.5–9.6)] (Figure 1; Table 2).

**Table 2:** Absolute risk of all-cause mortality.

Comorbidity	Absolute risk of all-cause mortality		
	1-year	2-year	3-year
Full population—age- and gender-matched			
TAVR	15.5% (11.9%–19.5%)	25.3% (20.4%–30.5%)	34.1% (27.8%–40.5%)
Background	0.8% (0.5%–1.3%)	1.7% (1.2%–2.4%)	2.9% (2.2%–3.9%)
Full population—age, gender and comorbidity matched			
TAVR	14.4% (10.8%–18.5%)	24.3% (19.2%–29.7%)	33.1% (26.6%–39.8%)
Background	2.9% (2.2%–3.8%)	5.1% (4.1%–6.2%)	7.5% (6.2%–8.9%)
Chronic heart failure			
TAVR	20.3% (12.6%–29.4%)	27.5% (25.9%–49.0%)	48.5% (34.1%–61.6%)
Background	12.1% (0.2%–15.5%)	19.1% (15.4%–23.1%)	26.8% (22.3%–31.5%)
Chronic obstructive pulmonary disease			
TAVR	21.0% (14.2%–28.7%)	29.6% (21.0%–38.7%)	35.9% (25.4%–46.5%)
Background	8.9% (6.9%–11.3%)	17.1% (14.2%–20.3%)	24.3% (20.7%–28.0%)
Chronic dialysis			
TAVR	25.4% (12.4%–40.8%)	43.2% (25.1%–60.1%)	50.3% (28.2%–68.8%)
Background	7.5% (4.2%–12.0%)	16.3% (11.1%–22.3%)	22.1% (15.7%–29.1%)
Prior cardiac surgery			
TAVR	10.7% (6.1%–16.8%)	26.6% (18.2%–35.7%)	32.6% (23.0%–42.6%)
Background	2.4% (1.4%–3.8%)	3.6% (2.3%–5.3%)	5.5% (3.8%–7.6%)
Prior stroke			
TAVR	27.6% (10.5%–47.9%)	34.8% (14.0%–56.8%)	34.8% (14.0%–56.8%)
Background	2.0% (0.4%–6.4%)	5.7% (2.1%–11.9%)	10.0% (4.6%–18.1%)
Prior Hodgkin's lymphoma			
TAVR	14.7% (4.1%–29.0%)	22.5% (9.4%–38.9%)	28.0% (12.2%–46.3%)
Background	0.6% (0.1%–3.1%)	2.6% (0.8%–6.0%)	2.6% (0.8%–6.0%)

Values are absolute risk of mortality (%) at 1, 2 and 3 years after TAVR/inclusion. TAVR, transcatheter aortic valve replacement.

### Subgroup analyses

**Figure 3:**
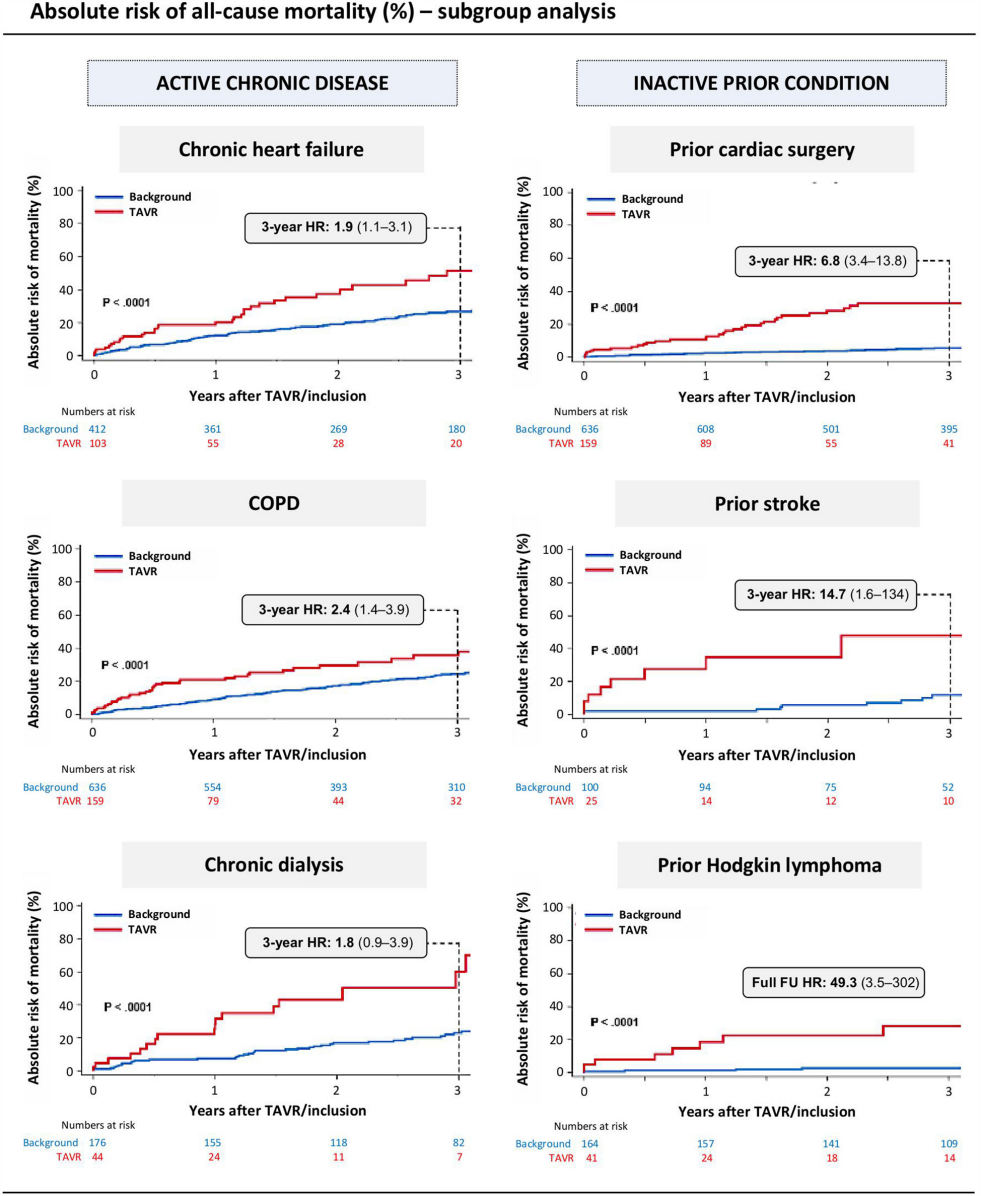
Absolute risk of mortality in subgroup analysis. For subgroup analysis, the cohort of patients undergoing TAVR with a specific comorbidity was matched and compared in a 1:4 ratio with age- and gender-matched individuals from the Danish National Registries with the same comorbidity. The primary outcome was all-cause mortality at 3 years. COPD, chronic obstructive pulmonary disease; FU, follow-up; HR, hazard ratio; TAVR, transcatheter aortic valve replacement.

Subgroup analyses of TAVR-treated patients with a concomitant, active chronic disease also demonstrated significantly higher 3-year all-cause mortality compared with matched controls. Specifically, patients undergoing TAVR with chronic heart failure, COPD and end-stage chronic kidney disease on dialysis had a 3-year all-cause mortality HR of 1.9, 2.4 and 1.8, respectively, as compared to their controls. Noteworthy, TAVR-treated patients with an inactive prior condition (prior cardiac surgery, stroke, and Hodgkin lymphoma) demonstrated an even greater differential increase in terms of all-cause mortality than their risk-matched controls, with a 3-year all-cause mortality HR of 6.8–24.7 as compared with their controls (Figure 3; Table 2).

## Discussion

Despite societal guidelines on valvular heart disease recommending SAVR as the first-line therapeutic choice for patients with severe symptomatic AS aged 65 years or under, young AS patients have been treated with TAVR in real-world clinical practice. The main findings of this multicenter study were: (1) the main reasons for TAVR preference were prior cardiac surgery, lung disease, frailty, reduced LV systolic function, and chronic renal failure; (2) all-cause mortality at 3 years was significantly higher in the TAVR-treated group compared with the control group when matched for age and gender and for age, gender and comorbidity; (3) the relative risk of all-cause mortality in TAVR-treated subgroups as compared to their controls was markedly higher for patients with an inactive prior condition—such as prior cardiac surgery, stroke or Hodgkin lymphoma—as compared to those patients with an active chronic disease.

### Young… but not suitable for cardiac surgery

SAVR is generally recommended for severe AS patients aged 65 years or under requiring intervention depending upon relative risk vs. benefit (1, 2). In case of high surgical risk, TAVR can be considered and has been used in this young patient population. However, the question is how and what determines high surgical risk or unsuitability for cardiac surgery. Standard surgical risk assessments are based on calculated risk scores, such as the STS risk score (4, 5). These risk scores are broadly determined by advanced age and are often less applicable to younger patients. Furthermore, the scoring systems do not capture all important comorbidities or recognize technical issues that can confront a surgeon, both of which can influence the Heart Team's decision-making and impact outcomes, rendering TAVR as the preferred treatment option for selected patients (6).

In the current study, more than two-thirds of young TAVR patients had 2 or more risk factors for SAVR related to baseline comorbidities (Figure 2). Not unexpectedly, prior cardiac surgery featured as a the most frequent reason to prefer TAVR due to the increased risk of repeated sternotomy. Similarly, difficulty in weaning from ventilation in the presence of severe COPD (7) or from cardio-pulmonary bypass in the presence of severe LV dysfunction can dissuade a surgeon from operating. Frailty is another patient comorbidity and reason to deny cardiac surgery for severe AS as it increases surgical morbidity and mortality (7). Chronic kidney disease with or without dialysis has also been associated with poorer outcomes post-cardiac surgery (8), whereas morbid obesity not only increases the risk of wound infection but also slows post-procedural recovery and rehabilitation (9). Also, the presence of a porcelain aorta is a major concern to surgeons who fear the increased risk associated with ascending aortic cannulation or cross-clamping (10). Finally, 8% of the TAVR cohort in this study were referred for TAVR predominantly based on patient preference.

### Safety and futility of TAVR in young multimorbid patients

The data collection in this multicenter study shows that TAVR is a safe procedure for young multi-morbid patients who are not suitable for open-heart surgery. Furthermore,the observed survival rates of 50%–70% at 3 years after TAVR support the vision that TAVR is not a futile treatment for this patient population. As a reminder, the natural history of untreated, severe AS is poor with a mean survival of 23 months and an 18% probability of survival at 5 years (11).

### Mortality outcomes in comparison with a risk-matched background population

The main purpose of this study was to compare medium-term mortality in this young multi-morbid TAVR population with a risk-matched background population without AS—and to investigate whether some subgroups had a better prognosis than others.

Overall, all-cause mortality at 3 years in the TAVR-treated group was 33.1% In comparison, the 3-year mortality rate after TAVR in the Evolut Low Risk Trial in patients at low surgical risk has been reported to be 6.3%—and this is in a TAVR population with a mean age of 74 years (12). Yet, in the YOUNG-TAVR Registry, in which patients ≤75 years undergoing TAVR had a higher mortality as compared to patients aged 76–86 years, but not compared with patients >86 years of age, attributable to the greater comorbidity burden in the youngest cohort (4). Similarly, the relatively high 3-year all-cause mortality (30%–35%) observed in our young TAVR cohort may be ascribed to the high comorbidity burden and indicates that these young, multi-morbid patients are biologically older than their age- and gender-matched controls.

In a subgroup analysis, the young TAVR patients with a specific primary comorbidity were compared with age- and gender-matched individuals with the same comorbidity. Two distinct patient cohorts were evaluated: patients with an active chronic disease—including chronic heart failure, COPD and chronic dialysis—and patients with an inactive prior condition, including prior cardiac surgery, stroke and curatively treated Hodgkin lymphoma. As expected, controls with an inactive prior condition had a much better prognosis than controls with an active chronic disease with 3-year mortality rates below 10% vs. 25%, respectively. On the other hand, the prognosis for TAVR patients with an inactive prior condition was not strikingly better compared with TAVR patients with a concomitant active chronic disease. This is also reflected by the much higher relative risk of all-cause mortality for TAVR-treated patients vs. controls in the cohorts with an inactive prior condition (HR, 6.8–24.7) as compared to a concomitant active chronic disease (HR, 1.8–2.4; Figure 3; Table 2).

In none of the subgroup analyses, TAVR treatment could restore life expectancy to the same level as observed for the age-, gender- and comorbidity-matched controls. In a previous study, we showed for a patient population with a mean age of 80 years and a low-to-intermediate risk profile, that TAVR can restore normal life expectancy for AS patients (13). In the same study, the relative risk (HR) of all-cause mortality for elderly TAVR patients with a high calculated surgical risk score was 1.9 as compared to an age- and gender-matched background population. This latter result is more in line with the current findings in our young patient cohort, indicating that this young multi-morbid TAVR cohort should be considered a ‘high risk’ population, and this is despite the lower calculated surgical risk scores. The reason(s) for the worse (than expected) survival prognosis in TAVR patients with prior cardiac surgery, stroke and Hodgkin lymphoma is not fully understood and remains a source of speculation. Nevertheless, this analysis provides interesting insights into the prognosis of multi-morbid young TAVR patients, which may be important in a multi-disciplinary Heart Team meeting and for the pre-procedural informative discussion with the patients and their relatives.

### Study limitations

There are several limitations to this study. Firstly, this is a retrospective, multicenter TAVR cohort study comparing a validated TAVR-treated population with an unvalidated control population from the Danish National Registries. Thereby, a heterogeneous TAVR patient population was compared with a homogeneous control population from Denmark, which may not be representative and comparable to the control populations in other participating countries. Another limitation is that the comparisons between TAVR and control cohorts in the subgroup analyses were performed matching for age, gender and a single primary comorbidity and not for multiple comorbidities, even though most patients were multi-morbid; this may have resulted in a relative risk over-estimation. Finally, we included patients treated with TAVR over a 10-year period during which time pre-procedural workup, TAVR devices and implantation techniques have improved, possibly resulting in better TAVR outcomes than were reported in the current study.

## Conclusions

Treatment of young, multimorbid AS patients aged ≤65 years with TAVR is safe and not futile—however, their all-cause mortality up to 3 years after TAVR is significantly higher compared with an age-, gender- and comorbidity-matched background population. Overall, the 3-year mortality rate in different TAVR-subgroups was high regardless of the underlying condition. Hence, a meticulous risk-benefit evaluation should be made when considering TAVR to treat these young AS patients.

## Data Availability

The raw data supporting the conclusions of this article will be made available by the authors, without undue reservation.
